# Investigation on the Role of Ionic Liquids in the Output Signal Produced by Bacterial Cellulose-Based Mechanoelectrical Transducers

**DOI:** 10.3390/s21041295

**Published:** 2021-02-11

**Authors:** Giovanna Di Pasquale, Salvatore Graziani, Santhosh Kurukunda, Antonino Pollicino, Carlo Trigona

**Affiliations:** 1 Dipartimento di Scienze Chimiche (DSC), University of Catania, Viale Andrea Doria 6, 95125 Catania, Italy; giovanna.dipasquale@unict.it; 2 Dipartimento di Ingegneria Elettrica Elettronica e Informatica (DIEEI), University of Catania, Viale Andrea Doria 6, 95125 Catania, Italy; santhosh.kurukunda@unict.it (S.K.); carlo.trigona@unict.it (C.T.); 3 Dipartimento di Ingegneria Civile e Architettura (DICAr), University of Catania, Viale Andrea Doria 6, 95125 Catania, Italy; apollicino@unict.it

**Keywords:** sustainable development, green sensors, deformation sensors, bacterial cellulose, ionic liquids, ionic electroactive polymers, open circuit voltage, short circuit current

## Abstract

Green sensors are required for the realization of a sustainable economy. Biopolymer-derived composites are a meaningful solution to such a needing. Bacterial Cellulose (BC) is a green biopolymer, with significant mechanical and electrical properties. BC-based composites have been proposed to realize generating mechanoelectrical transductors. The transductors consist of a sheet of BC, impregnated of Ionic Liquids (ILs), and covered with two layers of Conducting Polymer (CP) as the electrodes. Charges accumulate at the electrodes when the transductor is bent. Generating sensors can produce either Open Circuit (OC) voltage or Short Circuit (SC) current. In the paper, the OC voltage and SC current, generated from BC-based composites, in a cantilever configuration and subjected to dynamic deformation are compared. The influence of ILs in the transduction performance, both in the case of OC voltage and SC current is investigated. Experimental investigations of structural, chemical, and mechanoelectrical transduction properties, when the composite is dynamically bent, are performed. The mechanoelectrical investigation has been carried on both in the time and in the frequency domains. Reported results show that no relevant changes can be obtained because of the use of IL when the OC voltage is considered. On the contrary, dramatic changes are observed for the case of SC current, whose value increases by about two orders of magnitude.

## 1. Introduction

Networks of sensors (including wireless sensor networks) are required for the development of new ecologies such as, e.g., IoT, smart cities, Industry 4.0, precise agriculture. They can give a relevant contribution to the implementation of sustainable development, giving access to data required for more efficient exploitation of energy and raw materials. Unfortunately, technologies based on silicon electronics cannot cope with the needing for a circular economy, nor can produce green sensing systems. There is a needing for new technologies capable of giving a meaningful answer to such a request. Such technologies will be required to use renewable raw materials, save energy, and produce devices whose disposal does not negatively impact the environment. Both research and technological efforts are required for this aim.

Indeed, new technologies for the realization of sensors are required. Though, Electroactive Polymers (EAPs) have been the object of flourishing research in the last decades because of their unique transduction properties between the mechanical and electrical domains both as actuators [1] and sensors [2]. Sensing applications have been proposed, which use Ionic EAPs (IEAPs) [3,4,5,6,7]. Unfortunately, many IEAPs lack sustainability, both for the production process and the base materials. Most of them are, e.g., realized by using Nafion^TM^ or Flemion^TM^ [8], which are not considered green polymers [9,10,11,12]. Greener IEAPs can be obtained either by greener fabrication processes, which use less energy or a green chemistry approach yet using the same base materials [13] or by changing the starting raw materials, focusing on renewable and environment-friendly materials [11].

Bioderived polymers are among the most promising materials, both in terms of performance and environmental impact [14,15]. Among biopolymers, cellulose has been widely investigated for the realization of new electronics [16,17,18], and, more specifically of transducers [19,20,21]. A significant fraction of proposed cellulose-based applications deals with mechanical transducers. Both modifying [22,23,24] and generating piezoelectric sensors [25,26], have been proposed in the literature.

Bacterial Cellulose (BC) has recently raised the interest of researchers [27,28]. Though it shares the same chemical structure as plant-derived cellulose, it is a nanocellulose produced by *Acetobacter xylinum* bacteria in a suitable culturing environment. Due to its excellent mechanical properties, BC has been proposed as a reinforcing material [29,30], as well as for electronics [31]. BC-based composites have been proposed for the realization of both actuators [32], modifying [33,34], and generating [28] sensors.

BC–Ionic Liquid (BC–IL) composites have been already proposed in the literature for the realization of bending electromechanical transducers [32,35]. In those works, tri-layer structures, consisting of a BC-based bulk, infused with IL and covered with conductive electrodes are demonstrated to bend when a voltage input is applied at the electrodes.

Very few contributions exist on BC–IL composites as mechanoelectrical transducers [36].

Generating mechanoelectrical sensors are the topic of this paper. The transducer works in a cantilever configuration and an electrical signal is produced at the electrodes when it is bent. The cantilever configuration for sensing systems is widely adopted in the literature and many applications are reported, especially in the form of Microelectromechanical systems (MEMS) [37,38]. The discovery of IEAP mechanoelectrical transduction capability has further paved the road to the possibility of realizing IEAP-based transducers based on the cantilever configuration. More specifically, IEAP-based transducers in the cantilever configuration have been proposed both as sensors [5,39], and power harvesters [40].

The transducer, investigated in the following, consists of a BC-based layer, infused with ILs, and covered with two conducting electrodes. According to results already available for BC–IL actuators, a structure consisting of BC, infused with 1-Ethyl-3-methylimidazolium tetrafluoroborate (EMIM-BF_4_) as the IL, and covered with poly(3,4-ethylenedioxythiophene) polystyrene sulfonate (PEDOT-PSS), as Conducting Polymer (CP) is investigated. More specifically, EMIM-BF_4_ has been already proposed for the realization of actuators, being widely available in the market [32], though greener ILs [41], or even classes of solvents, such as Deep Eutectic Solvents (DES) could be investigated to this aim, as environmentally friendly solvents [42,43,44,45]. In the same way, electronic conductive layers other than CPs, such as carbon-based layers [46] could be used for the electrodes. Nevertheless, the focus of this paper is to investigate BC-based composites that already have been demonstrated as electromechanical actuators.

The Authors have investigated the mechanoelectrical transduction capabilities of BC-based composites, infused with ILs to realize EAPs [36,47]. Additionally, applications exploiting either non-bioderived polymers and bioderived polymers have been proposed, giving evidence of the interest in the development of new greener technologies for sensor fabrication [48,49].

In [50], the Authors have already investigated the role of ILs in the mechanoelectrical transduction capabilities of such composites, named in the following BC–IL-CPs. In that work, a temperature-controlled chamber was used and the current produced at the PEDOT-PSS electrodes, when dynamically deformed, was recorded. Experiments were run at different temperature values. It was observed that the magnitude of the produced signal depended on the working temperature and reached a constant minimum value when working temperature values lower than the IL melting point, i.e., 15 °C for EMIM-BF_4_, were imposed in the chamber. This demonstrated the active role of the IL in the composite transduction capability. Nevertheless, no quantitative investigation was performed on the nature of the produced current. In the present paper, the role of the IL in the quality of the produced signal is investigated. To further elucidate the contribution of ILs in the mechanoelectrical transduction capabilities of this class of composites, we experimentally compare the performance of BC-based mechanoelectrical transducers as vibration motion sensors, when in a cantilever configuration. BC-based compounds, without any infused IL or with the infusion of an IL are considered. More specifically, in a first set of experiments, we investigated the output signal amplitude as a function of the input amplitude to gain information on the sensor linearity. In a second investigation, the produced output signals were analyzed in the frequency domain, to investigate the noise and distortion contributions in the sensor output. Both results can give useful information when the possibility of using the composite as a sensor is of interest.

Since the core of the composite is also a dielectric, accumulated charges produce a corresponding voltage at the electrodes, when in open circuit (OC) conditions, or a flowing current if the electrodes are connected in short circuit (SC) conditions [51]. In the first case, the relevant signal is the voltage generated at the compound electrodes, while in the second case, the flowing current is of interest.

The paper investigates both the structural properties of the composites and their mechanoelectrical transduction properties. More specifically, the performance of the composite by using both the conditioning approaches, when dynamically deformed are investigated. Sinusoidal signals of variable amplitude have been considered and an investigation has been performed on the time records of the output produced signals. Then, an in-deep analysis has been performed on the signals produced in correspondence with the composite mechanical resonance frequency. Finally, the SIgnal-to-Noise And Distortion ratio (SINAD) has been used as a further measure of the effect of the IL presence, both on OC and SC conditioning conditions.

## 2. Materials and Methods

### 2.1. The Bacterial Cellulose and the Composites

BC was kindly provided by BioFaber^®^. Sheets of dehydrated BC (approximatively 395 μm thick) were preliminarily dried in an oven at 65 °C for 24 h just before use. For the case of BC–IL composites, a BC strip (5 × 5 cm) was soaked with IL (EMIM-BF_4_—by Sigma Aldrich, USA) in a petri dish for 24 h in a desiccator containing anhydrous CaCl_2_. Then the composite membrane was kept in an oven at 65 °C for 24 h. The amount of EMIM-BF_4_ absorbed by BC was 34% by weight.

Both BC and BC–IL composite-based devices were fabricated by coating the membranes with a CP (PEDOT-PSS—1.3 wt% dispersion in water, Baytron P AG) on both sides via a film spreader 24 μm thick and drying the obtained sample in an oven at 65 °C for 5 min. This procedure was repeated four times to obtain continuous and homogeneous electrodes. In the end, the total amount of CP and IL present in BC–IL-CP, was estimated as:

(1) $$CP\;weight\;\%=\;\frac{W_{Dry\;BC-CP}-W_{Dry\;BC}}{W_{Dry\;BC}}\times 100$$

(2) $$IL\;weight\;\%=\;\frac{W_{Dry\;BC-IL-CP}-W_{Dry\;BC}}{W_{Dry\;BC}}\times 100-CP\;weight\;\%$$

**Figure 1:**
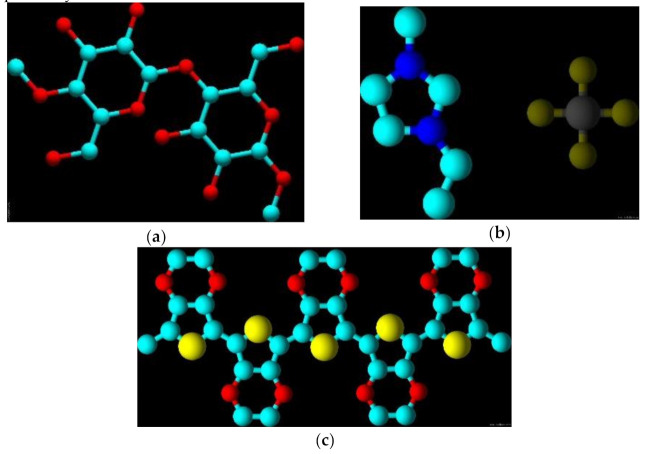
3D chemical structure of BC (**a**), EMIM-BF_4_ (**b**), and PEDOT (**c**).

The chemical structures of BC, EMIM-BF_4_, and PEDOT are shown in Figure 1a–c, respectively.

### 2.2. Structure and Chemical Characterization

Scanning Electron Microscopy (SEM) micrographs have been obtained using a Cambridge 90 instrument, equipped with an energy dispersive X-ray microanalysis (EDX) facility. Fourier Transform IR (FTIR) analysis was performed by a PerkinElmer Spectrum 100 spectrometer, at room temperature, from 4000 to 650 cm^−1^, with a resolution of 2.0 cm^−1^. A universal Attenuated Total Reflectance (ATR) sampling accessory was used for the measurements, which were made directly on samples, without any preliminary treatment. ThermoGravimetric Analyses (TGA) have been carried out by a Shimadzu model DTG-60 instrument. TGA curves have been recorded at a heating rate of 10 °C min^−1^, under static air atmosphere, from 35 to 700 °C. Analyzed sample mass varied between 8.0 and 11.0 mg.

### 2.3. Mechanoelectrical Transduction

**Figure 2:**
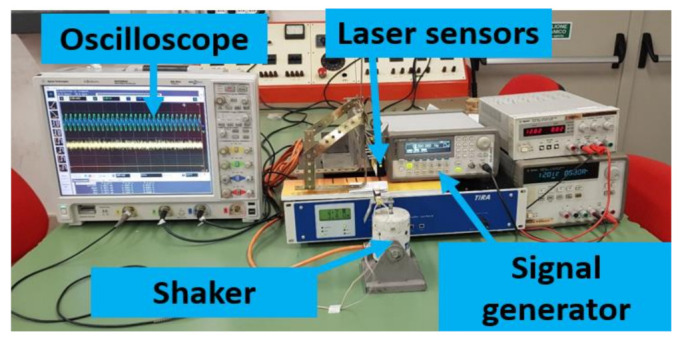
Experimental setup, showing the shaker used and related driving system (Signal generator and amplifier), the laser sensors for the composite deflection estimation, and the oscilloscope, for the recording of signals produced both by the laser sensors and by the mechanoelectrical BC-based transducer.

To investigate the BC-based transducers, a suitable experimental setup was realized as shown in Figure 2. In particular, it is composed of:

- A shaker TIRA-vib S503 connected with an amplifier used to impose mechanical vibrations.
- A signal generator Agilent 33220A was used to drive the amplifier imposing a suitable waveform.
- We used two laser sensors, with power suppliers, to measure the displacement at the tip and the anchor of the BC-based transducer. The deformation of the device was estimated by the difference between the measurements provided by the laser sensors.
- An Agilent Technologies digital oscilloscope (MSO906A), used to acquire the output of the device and the laser sensors signals.

**Figure 3:**
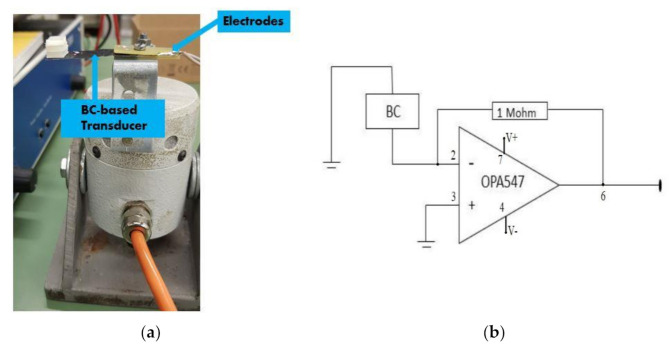
(**a**) Zoom of the BC-based transducer mounted on the shaker. In the figure, the rigid electrical contacts can be seen; (**b**) electrical scheme of the conditioning circuitry used for the current to voltage conversion, in SC working conditions.

The measurements were performed considering OC voltages and SC currents for BC-based transducers in both the absence and the presence of ILs. For this reason, a current to voltage converter was used for the SC configuration. Figure 3a shows a zoom of a device in the cantilever beam configuration with a proof mass at the tip to increase the sensitivity of the oscillator. The picture includes the electrical contacts used to connect the BC-transducer. Figure 3b shows a schematic of the electrical circuitry used for SC conditioning. A feedback resistor equal to 1 MΩ was used in this case. In the case of OC conditioning, the direct connection with the oscilloscope was used, instead.

**Figure 4:**
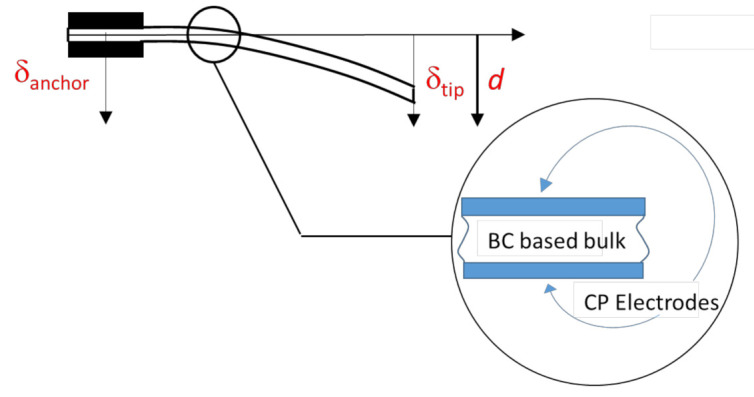
A scheme of the BC composite deformation *d*, along with the reference directions for the anchor and tip displacements.

The two laser sensors pointed at the anchor and the free end of the composite, in the cantilever configuration. The voltage signals produced by the laser sensors were converted into the corresponding motion values from the reference configuration. The obtained signals were finally, processed to estimate the beam deformation. A scheme reporting the reference directions is given in Figure 4, while in (3) the expression of the deflection *d* is given.

(3) $$d=\left(\delta_{tip}-\delta_{anchor}\right)=k\left(V_{tip}-V_{anchor}\right)$$

where

- $\delta_{tip}$ and $\delta_{anchor}$ are the displacements at the tip and the anchor, respectively.
- *k* = 0.8 mm/V is the laser sensors transduction constant.
- $V_{tip}$ and $V_{anchor}$ are the laser sensors’ output signals, in volts, increasing for increasing values of the displacements. Both voltage values are subtracted for the corresponding values at the mechanical equilibrium positions, assumed as the reference.

The deformation imposed on the composite produces a charge accumulation at the CP electrodes. A couple of rigid electrodes is used both to collect the electrical signal and to fix the base of the transducer to the shaker shaft, in a cantilever configuration.

Finally, the SINAD, which is commonly adopted to characterize transmission systems, ADC, and DAC converters, is estimated in the paper to get a further indication of the role of the IL in the mechanoelectrical capabilities of the BC-based composites, according to the used conditioning approach. SINAD can be defined as the desired signal power times any undesired component power. The ratio is generally expressed in decibels:

(4) $$SINAD=10\;log_{10}\frac{S}{N+D}$$

where *S* is the signal power. *N* is the noise power. *D* is the distortion contribution.

## 3. Results

### 3.1. CP Content and IL Uptake

The total amount of CP and IL present in BC–IL-CP, were computed according to Equations (1) and (2), respectively. The CP content resulted in approximatively 2% by weight. The IL uptake was evaluated to be about 9% by weight.

### 3.2. Structure and Chemical Characterization of BC-CP and BC-IL-CP Device

**Figure 5:**
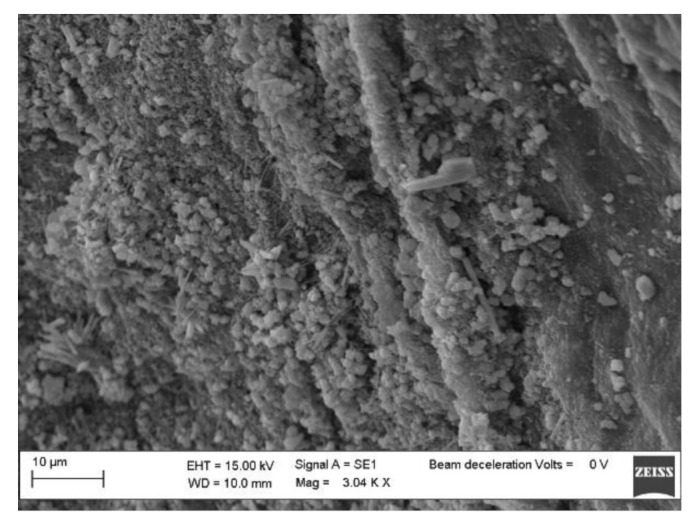
SEM micrograph of the BC surface.

**Figure 6:**
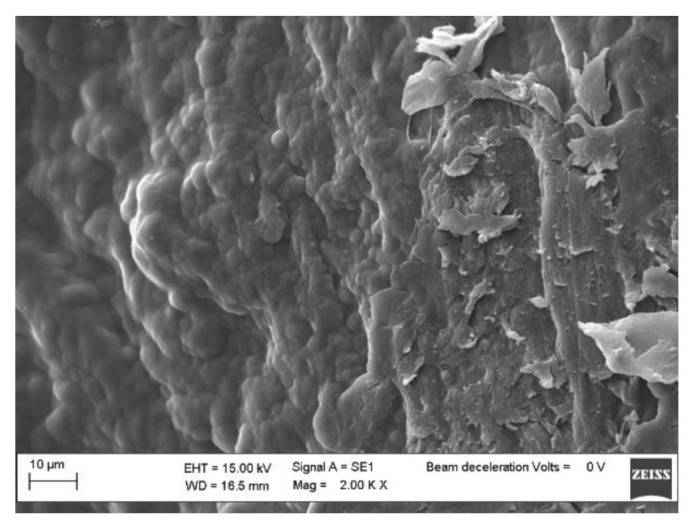
SEM micrograph of the BC-CP surface.

**Figure 7:**
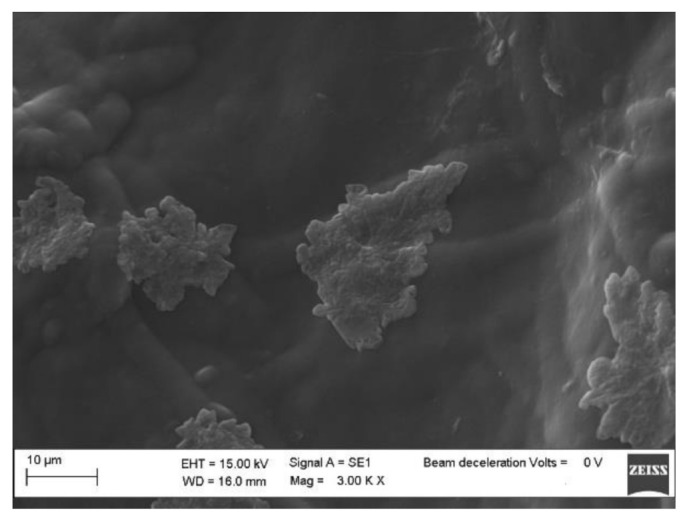
SEM micrograph of the BC–IL-CP surface.

The morphologies of pure BC, BC-CP, and BC–IL-CP membranes were investigated by SEM. Results are reported in Figure 5, Figure 6 and Figure 7.

**Figure 8:**
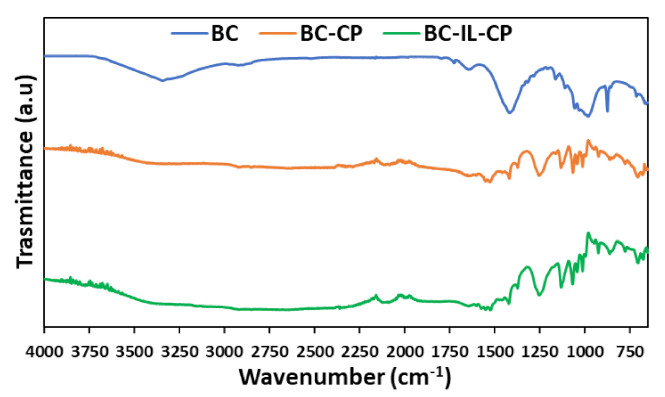
ATR-FTIR spectra of BC, BC-CP, and BC–IL-CP.

**Figure 9:**
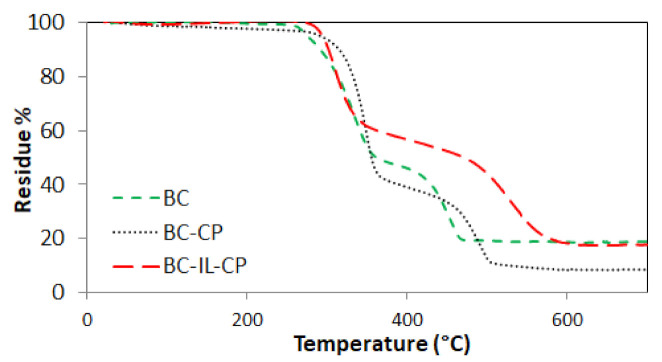
TGA curves of BC, BC-CP, and BC–IL-CP.

Figure 8 reports the ATR-FTIR spectra of BC, BC-CP, and BC–IL-CP, in the wavenumber 4000 to about 450 cm^−1^. Finally, Figure 9 reports the results of the TGA, in the range 0 to about 700 °C, for the same composites.

### 3.3. Mechanoelectrical Transduction

Cantilever sensing systems can be used at their mechanical resonant frequency when a signal of the maximum level can be obtained [36,52,53,54].

**Figure 10:**
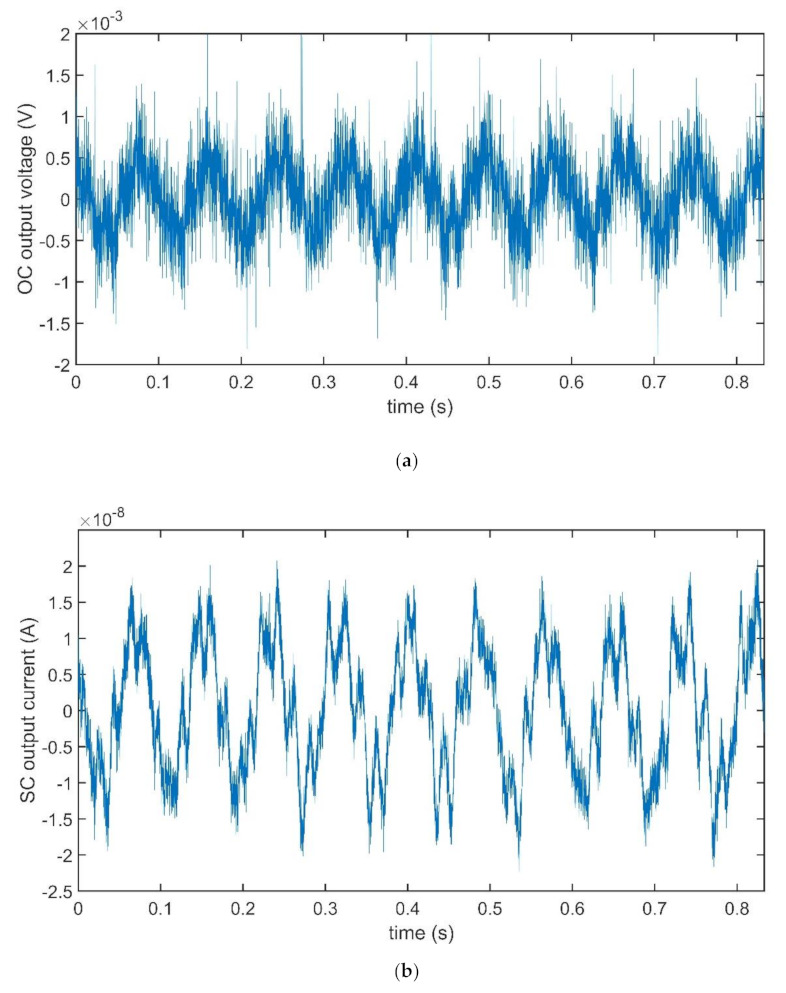
Time plots of OC voltage (**a**) and SC current (**b**) for the BC-CP composite. Both records were obtained by imposing a sinusoidal vibrating at the composite anchored end. The frequency of the input was fixed at 12 Hz.

The investigation was performed by using the sinusoidal response of the devices and a preliminary investigation was run to determine the mechanical resonant frequency of the devices. Time records of obtained OC voltage and SC current are reported in Figure 10a,b, respectively, for the case of the BC-CP composite, when subjected to a sinusoidal anchor vibration, with a frequency equal to 12 Hz, close to the composite mechanical resonance frequency, and maximum deformation equal to about 3.5 mm.

**Figure 11:**
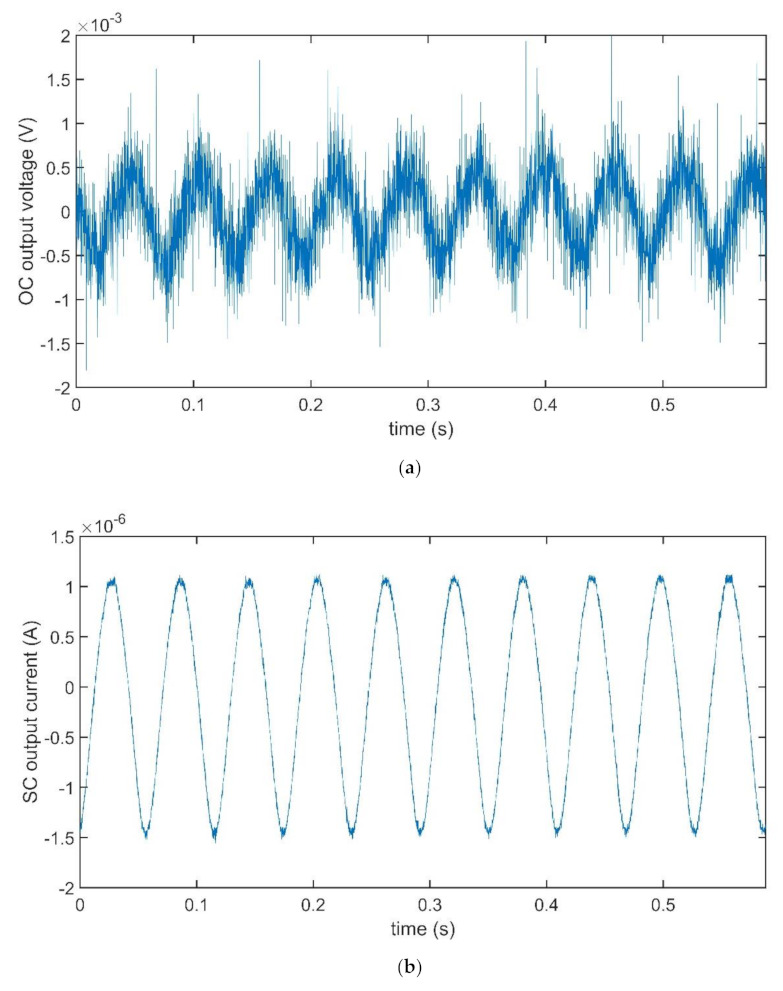
Time plots of OC voltage (**a**) and SC current (**b**) for the BC–IL-CP composite. A sinusoidal vibrating input, at the frequency of the input, was fixed at 12 Hz, was applied to the anchored end of the composite.

The time recordings obtained for the case of the BC–IL-CP are reported in Figure 11a,b, respectively, for the matter of comparison. Both signals are acquired at 17 Hz, corresponding to the mechanical resonance frequency of the composite.

It is quite evident that small differences can be observed for the amplitude of the OC voltage signals, regardless of the presence of ILs in the composites. On the contrary, the amplitudes of the SC currents change dramatically, with an increase of about two orders of magnitude. Starting from the significant changes outlined above, a deeper comparison was performed aimed at outlining the differences in the produced output signals.

**Figure 12:**
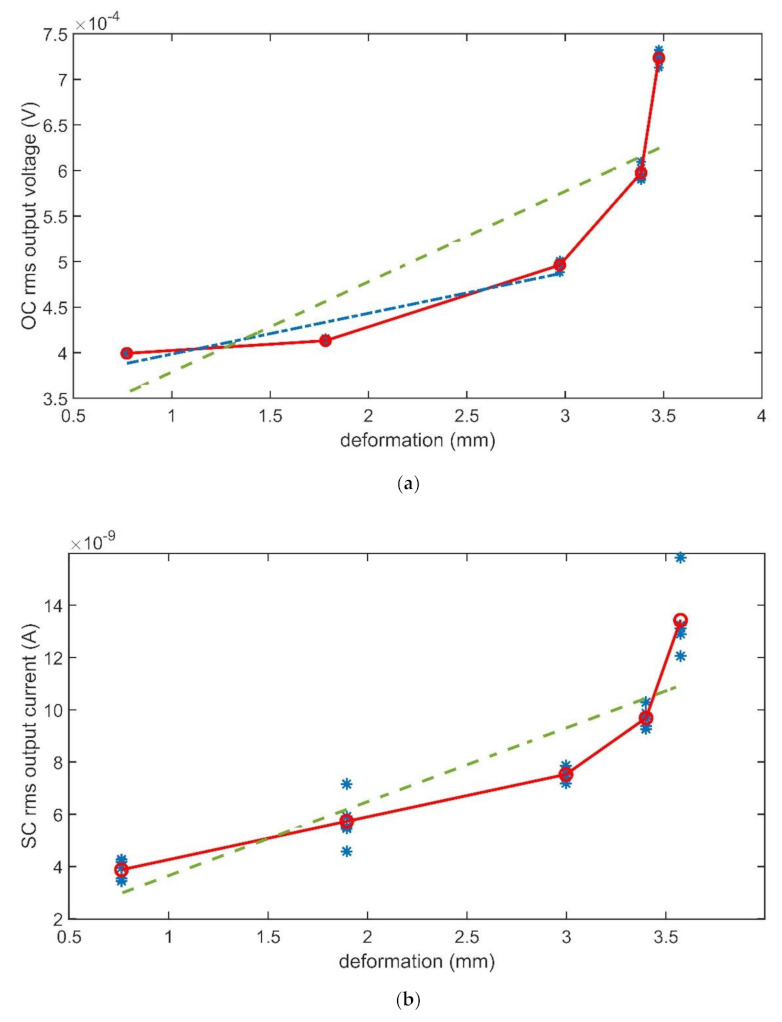
Rms values of the BC-CP OC voltage (**a**) and SC current (**b**) at the composite mechanical resonance frequency (12 Hz) as a function of the deformation.

**Figure 13:**
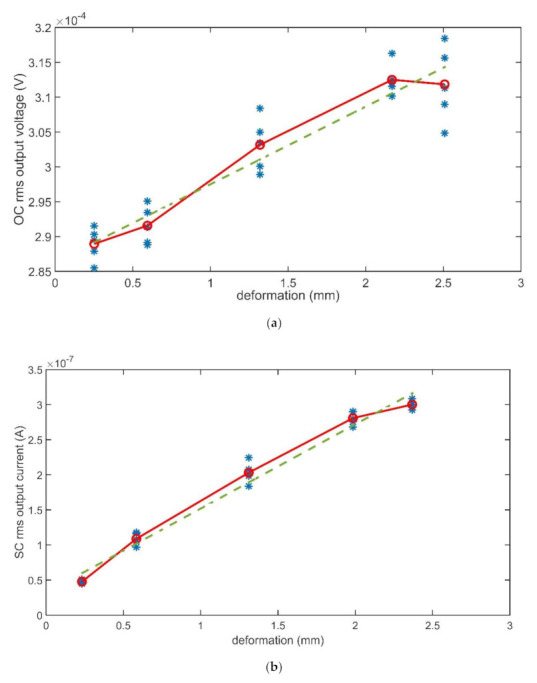
Rms values of the BC-IL-CP OC voltage (**a**) and SC current (**b**) at the composite mechanical resonance frequency (12 Hz), as a function of the deformation.

The composites were forced by input mechanical signals with different amplitudes. Then, the changes in the output signal amplitude were investigated. This analysis was performed at the composites’ mechanical resonant frequencies. In total, five different values were considered for the deformation. Results are reported in Figure 12 and Figure 13, which show the rms values of the output signals (OC voltage and SC current, respectively) as a function of the input applied deformation, estimated by using (3). A total of five repeated observations were performed for each value of the deformation. The differences in the values reported in the deformation axes in Figure 12and Figure 13are due to the difficulty of imposing a given deformation. The controlled input was the anchor motion, while the deformation was produced by the beam inertia.

The solid lines, which connect the mean values of the output signal as a function of the deformation value, have been added for the help of the eye. The green dashed lines are obtained by regressing the mean values referenced above. The meaning of the dash-dotted blue line in Figure 12a will be discussed in the next section.

**Figure 14:**
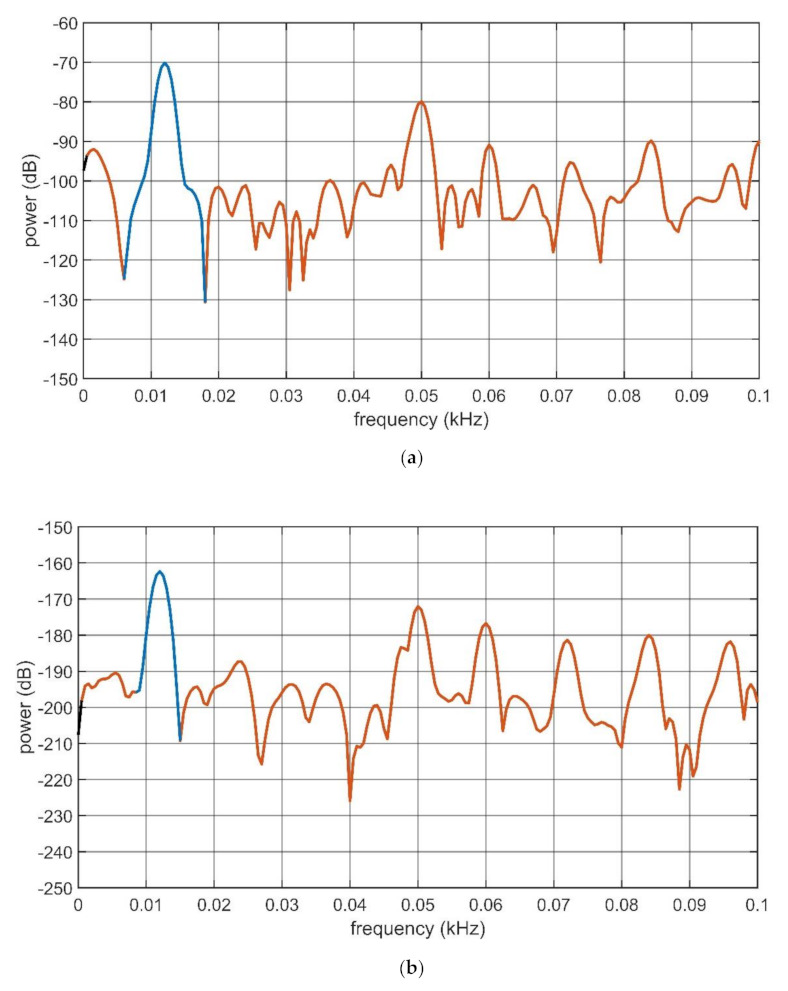
Power spectra of the BC-CP OC voltage (**a**) and SC current (**b**) for the transducer forced at its mechanical resonance frequency (12 Hz).

**Figure 15:**
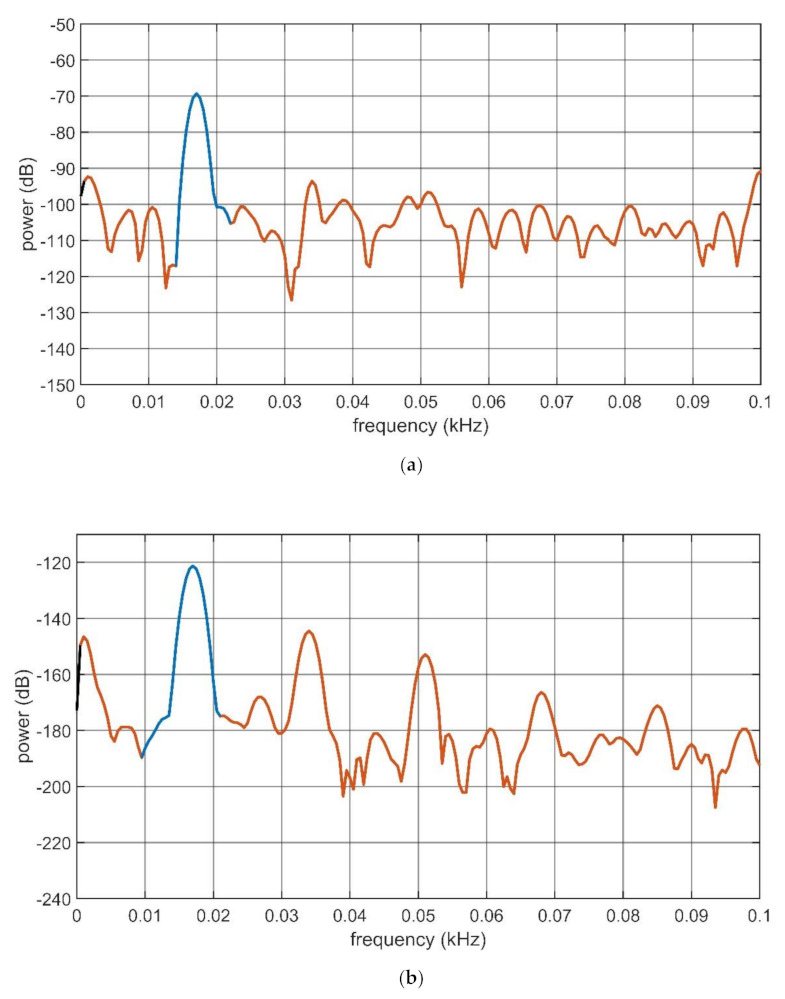
Power spectra of the BC-IL-CP OC voltage (**a**) and SC current (**b**) for the transducer forced at its mechanical resonance frequency (17 Hz).

Finally, the SINAD, see (4), was estimated as an indication of the IL contribution to the mechanoelectrical transduction capabilities of both the composites. All the signals were acquired at 10^4^ s^−1^. The SINAD value was computed for both the composites in OC and SC conditioning conditions so that four different values were obtained. The same signals used to trace the graphs reported in Figure 10 and Figure 11 were used for estimating the SINAD value for each configuration. The SINAD was estimated by using Matlab^®^. The low-frequency portion of the power spectra produced by Matlab^®^ is shown in Figure 14 and Figure 15, respectively. Estimations of the SINAD were performed at the mechanical resonance frequency of the transducers. In the computation, the contribution of the DC component is not considered. The blue sections of the curves reported in the mentioned figures indicate the fundamental frequencies of the recorded signals (i.e., the resonant mechanical frequencies of the transducers, for the considered cases). The red section indicates data that contribute to the noise and distortion computation. The obtained SINAD, estimations are reported in Table 1.

## 4. Discussion

The SEM micrographs of the sample surfaces show that, after CP coating on BC, the shape of globular BC agglomerates (Figure 5) can be still distinguished although the PEDOT deposition produced a regular morphology (Figure 6). The surface morphology of the BC-IL-CP composite is even smoother (Figure 7) with island features mainly constituted of IL as indicated by the composition information obtained from EDX.

The good surface coverage by CP is testified by spectra and TGA. While in ATR FTIR BC spectrum the main bands (Figure 8) can be assigned to cellulose functional groups: 3000–3600 cm^−1^ (O-H stretching), 2920–2880 cm^−1^ (aliphatic C-H stretching), 1640 cm^−1^ (bending of absorbed H-O-H), 1424 cm^−1^ (in-plane bending of H-C-H groups) and 1110–874 cm^−1^ (asymmetric stretching of the ether C-O-C bond and C-OH groups stretching), the spectra of the BC-CP and BC-IL-CP devices shows almost the same signals mainly arising from the PEDOT thiophene ring: i.e., 1576 cm^−1^ (C=C), 1254 cm^−1^ (C-C) and 948 cm^−1^ (S-O).

We further explored the thermal stability of the BC-IL-CP membrane by using TGA, demonstrating that the continuous CP electrodes act as a barrier (Figure 9). The TGA curve of pure BC showed three decomposition stages. The first step, in the range 80 to 150 °C, was attributed to dehydration and fragmentation of polymer chains. The second stage is associated with a maximum degradation rate at 336 °C, due to the formation of a hydromonosaccharide and conversion into low molecular weight polysaccharides. The third step is associated with a maximum degradation rate at 446 °C, corresponded to the formation of charred degradation products.

The presence of the electrode promoted the curve shifting towards higher temperatures, especially notable in the case of the third degradation step, confirming that the electrodes act as an obstacle to the evolution of the volatile degradation by-products. In the case of BC-IL-CP, this effect is less pronounced during the second degradation step because of the presence of the IL that disrupts most of the BC crystalline domains.

Looking at results reported in Figure 12 and Figure 13, as a first difference, it is quite evident that, in the absence of the IL, both the produced OC voltage and SC current show strong nonlinearities, while, in the presence of the IL, the composite shows better linearity. The regression lines are, in fact, unable to fit data in Figure 12, while fit quite well data in Figure 13.

A second difference emerges if the changes in the output signals as a function of the input changes are considered. To this aim, the slopes of the regressing lines for data reported in Figure 12 and Figure 13, were considered. Though the obtained lines are in no way to be considered as an attempt to produce the characteristic lines of the transducers, the slopes can give significant insight into how deep the change is induced by the presence of the ILs. The slopes obtained in the four investigated cases are reported in Table 2.

Values reported in the table show that the slope of the line decreases by about one order of magnitude because of the presence of the IL in the OC voltage signal. For the matter of a better comparison, a second regression line was determined for data reported in Figure 12, indicated with the blue dash-dotted line in the figure. This second line was obtained limiting considered data in such a way that the investigated deformation range is about the same for the cases of BC-CP and BC-IL-CP composites. In this latter case, the slopes of the CB-CP composite, reported in round brackets in Table 1, results closer to that of the BC-IL-CP composite. The influence of the IL presence in the BC-based composites is more evident when the SC current is considered. The slope of the line referring to the SC current increases by about two orders of magnitude when the IL is infused in the composite.

Results reported in Figure 14 and Figure 15, and in Table 2 show that the infusion of the IL produces a linearization of the input-output relationships of the composites. Moreover, the SC current conditioning is greatly enhanced by the presence of the IL. Results reported for the SINAD estimation (see Table 1) confirm that a moderate change is produced, for the case of the OC voltage. Dramatic differences occur when the SC generated current is considered. More specifically, in this second case, the SINAD increases by about 16 dB, i.e., about 40 times, when the composite containing the IL is considered.

Both the possibility of working at the sensor resonant frequency and the reduction in the noise level, obtained when using the ILs, especially, in the case of the SC current configuration, has a beneficial effect on the output signal. More specifically, the lower the noise contribution, the smaller the level of the input signal, i.e., deformation for the considered sensor, that can be sensed. This is useful information when the resolution of the sensor is of interest.

To the best of the Author’s knowledge, this is the first report referring to the influence of both the IL presence and conditioning circuitry in the sensing capabilities of BC-based composited as generating mechanoelectrical transducers. Though preliminary results are reported for similar configurations in [15,55], only the case of OC voltage generation is reported. Moreover, in both cases, different base-materials are considered. In [15], chitosan is used as the bulk and it is infused EMIM-BF_4_. In [55], PVDF is used as the bulk, containing the electrolyte and polypyrrole based electrodes. Different electrolytes were used to study the influence of the cation on mechanoelectrical transduction. The OC peak-to-peak voltage was of the order of 0.28 mV, in [55], and raised to 13.56 mV, in [15].

Finally, the reported results are to be intended as preliminary for the characterization of the prosed BC-based sensor as a generating deflection sensor mounted in a cantilever configuration and working at its resonant frequency. Further investigations are required to better understand and model the physics that rules the transduction phenomena, as well as the relationship that links the composite deformation to the produced signal. Attention needs to be paid to the consequences of the conditioning approach, i.e., OC or SC, to the useful working frequency range for the composite as a mechanical sensor. More specifically, while IEAPs are reported to be capable of sensing quasi-static deformations in OC conditions, SC conditioning introduces a derivative effect, so that any capability of sensing such signals is hindered [56]. Both the nature of the input quantity and the quality of the produced output signal can suggest using either OC or SC conditioning circuits.

## 5. Conclusions

The paper investigates BC-based mechanoelectrical transducers as deformation sensors. The cantilever configuration, widely adopted for MEMS and IEAPs, is considered for BC-based sensors. More specifically, the role of ILs absorption in the generating mechanoelectrical transduction of BC-based composites is investigated. Composites without any IL or infused with an IL are considered to this aim. Both OC voltage and SC current generated by the BC-based composites are investigated. The experimental analysis has been run of the structural, chemical, and mechanoelectrical transduction capabilities of the composites, in the absence of the IL and in its presence.

Reported results show that the conditioning approach greatly influences the performance of the composites mechanoelectrical transducers. This is a relevant result when designing sensing systems based on BC. More specifically, it emerged that, while a modest difference emerges if the OC voltage is considered, the characteristic of the SC current changes dramatically. The amplitude of the produced signals increases by about two orders of magnitude. This result is confirmed if the SINAD in the presence of a sinusoidal signal is investigated. The SINAD value for the SC current signal in the presence of ILs is about 16 dB larger, i.e., about 40 times, larger than the SINAD of the signal produced by the BC composites, without any IL.

This is an important result when considering the practical sensing applications of this new class of transducers. The designer can get a significant insight into the best conditioning approach that, in turn, can result in better-performing applications.

The reported results are to be intended as preliminary. Further investigations are required to model the phenomena that rule the transduction capability of this class of BC-based composites. The influence of the nature of the specific ILs used in the composite on its transduction capabilities need also to be investigated.

The availability of different ILs, including bio-derived ILs, or DES, as candidates for the realization of BC-based sensors, and corresponding models can greatly improve the practical relevance of this new class of green deformation sensors. They can represent tools for designing meaningful sensing applications.

## Figures and Tables

**Table 1:** SINAD Values for BC-Based Composites.

	BC-CP	BC-IL-CP
**OC voltage (dB)**	−0.79	0.72
**SC current (dB)**	5	20.87

**Table 2:** Slopes of the linear regression for data reported in Figure 12 and Figure 13.

	**BC-CP**	**BC-IL-CP**
**OC voltage (V/mm)**	9.92 × 10^−5^ (4.48 × 10^−5^)	1.11 × 10^−5^
**SC current (A/mm)**	2.80 × 10^−9^	1.19 × 10^−7^

## Data Availability

Not applicable.
